# Regional hyperthermia and moderately dose-escalated salvage radiotherapy for recurrent prostate cancer. Protocol of a phase II trial

**DOI:** 10.1186/s13014-015-0442-4

**Published:** 2015-07-08

**Authors:** Arndt-Christian Müller, Daniel Zips, Vanessa Heinrich, Ulf Lamprecht, Otilia Voigt, Susen Burock, Volker Budach, Peter Wust, Pirus Ghadjar

**Affiliations:** Department of Radiation Oncology, Eberhard Karls University, Hoppe-Seyler-Str. 3, 72076 Tübingen, Germany; Department of Radiation Oncology, Charité Universitätsmedizin Berlin, Augustenburger Platz 1, 13353 Berlin, Germany

**Keywords:** Hyperthermia, Postoperative, Prostate cancer, Prostatectomy, Salvage radiotherapy, Study protocol

## Abstract

**Background:**

Current studies on salvage radiotherapy (sRT) investigate timing, dose-escalation and anti-hormonal treatment (ADT) for recurrent prostate cancer. These approaches could either be limited by radiation-related susceptibility of the anastomosis or by suspected side-effects of long-term ADT. A phase II protocol was developed to investigate the benefit and tolerability of regional hyperthermia with moderately dose-escalated radiotherapy.

**Methods:**

The study hypothesis is that radio-thermotherapy is a safe and feasible salvage treatment modality. The primary endpoint is safety measured by frequency of grade 3+ genitourinary (GU) and gastrointestinal (GI) adverse events (AE) according to Common Toxicity Criteria (CTC) version 4. Feasibility is defined by number of hyperthermia treatments (*n* ≥ 7) and feasibility of radiotherapy according to protocol. Target volume delineation is performed according to the EORTC guidelines. Radiation treatment is administered with single doses of 2 Gy 5×/week to a total dose of 70 Gy. Regional hyperthermia is given 2×/week to a total of 10 treatments.

**Results:**

European centres participate in the phase II trial using intensity modulated RT (IMRT) or volumetric modulated arc technique (VMAT). The initiating centres were participants of the SAKK 09/10 study, where the same patient criteria and target volume definition (mandatory successful performed dummy run) were applied insuring a high standardisation of the study procedures.

**Conclusions:**

The introduced phase II study implements highly precise image-guided radiotherapy and regional hyperthermia. If the phase II study is found to be safe and feasible, a multicenter phase III study is planned to test whether the addition of regional hyperthermia to dose-intensified sRT improves biochemical control.

## Background

### Salvage radiotherapy for biochemical recurrence after prostatectomy

Approximately 15–40 % of patients experience a biochemical relapse after radical prostatectomy within 5 years [1, 2]. Salvage radiotherapy (sRT) improves cancer-specific survival in these patients compared to observation alone [3]. Therefore, early salvage treatment at PSA levels below 0.5 ng/ml is recommended by European guidelines [4] to ensure a benefit for more than half the patients [5, 6]. In addition, a short prostate-specific antigen doubling time of <6 months is prognostic for metastasis suggesting only little benefit of sRT [7]. A steep sigmoidal dose–response curve was detected in the radiation dose range between 60–70 Gy. Current dose escalation from 60 to 70 Gy would increase biochemical control by approximately 20 %, indicating that dose-escalation is beneficial for these patients [6].

### Recruiting and unpublished randomized studies on salvage radiotherapy

Ongoing and/or up to now unpublished randomized studies on sRT to the prostatic fossa investigate timing [8], dose-escalation [9] and anti-hormonal treatment (ADT) [10, 11]. However, all salvage optimization studies have a risk of potentially increased morbidity, either due to radiation susceptibility of the anastomosis, or due to pharmacological side effects related to long-term ADT. The first analysis of toxicity from the dose-escalation trial comparing 64 Gy with 70 Gy demonstrated no significant differences between both dose levels with regard to GU and GI acute toxicity scored with CTCAE version 4 [12]. Therefore, further treatment intensification is reasonable and might be performed without or with acceptable increased side effects.

### Rationale for this trial

Regional hyperthermia is successfully applied as a response modifier with radiotherapy, leading to improved local control in different tumour entities including rectal cancer [13–16], gynaecologic malignancies [16–18], sarcoma [19, 20] and others. In prostate cancer, radio-thermotherapy also increased local tumour control in advanced disease with low toxicity rates [21–27]. Quality of life was not affected by regional hyperthermia [27]. According to a recently performed estimation, addition of hyperthermia to radiotherapy in prostate cancer would result in a dose-escalation equivalent to at least 10 Gy [28]. Consequently, application of regional hyperthermia could be a method to avoid radiation-based dose-escalation above 70 Gy and to circumvent ADT-related toxicity in prostate cancer patients after surgery.

There is limited experience with radio-thermotherapy as a salvage modality in prostate cancer [23] and a phase II protocol with an interim safety analysis was therefore developed. The phase II study investigates the potential benefit and tolerability of hyperthermia with moderately dose-escalated salvage radiotherapy to a total dose of 70 Gy in biochemically recurred prostate cancer after prostatectomy.

## Methods and design

### Study design

**Fig. 1:**
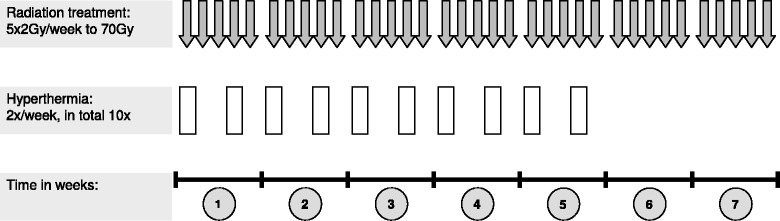
Design of the phase II study of radio-thermotherapy for biochemical recurrent prostate cancer

The study is designed as a multicentre prospective phase II study. An outline of the study procedures is given in Fig. 1.

### Study hypothesis

The study hypothesis is that radio-thermotherapy is a safe and feasible salvage treatment modality.

### Primary endpoint

The primary endpoint is safety measured by frequency of grade 3+ genitourinary (GU) and gastrointestinal (GI) adverse events (AE) according to CTCAE version 4 [29]. Feasibility is defined as the number of hyperthermia treatments (*n* ≥ 7) and feasibility of radiotherapy according to protocol in ≥ 95 % of patients.

### Secondary endpoints

1. Quality of life (QoL) assessment
2. Biochemical progression-free survival defined as PSA-rise > 0.4 ng/ml or increasing PSA-level where the initial PSA-level is above 0.4 ng/ml.
3. Clinical progression-free survival.
4. Time without ADT, i.e., time until initiation of ADT.

### Assessment of QoL

Quality of life (QoL) assessment using the EORTC QLQ-C30 [30], the QLQ-PR25 [31] and the Memorial Anxiety Scale for Prostate Cancer (MAX-PC) [32] questionnaires is performed at 3, 12, 24, and 36 months.

### Inclusion criteria

Patients with the following characteristics will be eligible for this study.

1. Lymph node negative adenocarcinoma of the prostate treated with radical prostatectomy at least 12 weeks before randomization. Tumour stage pT2a-3b, R0-1, pN0 or cN0 according to the UICC TNM 2009; Gleason score available.
2. PSA progression after prostatectomy defined as two consecutive rises with the final PSA > 0.1 ng/ml or three consecutive rises. The first value must be measured at least 4 weeks after radical prostatectomy.
3. PSA at randomization ≤ 2 ng/ml.
4. No evidence of macroscopic local recurrence or metastatic disease on pre-sRT-MRI (magnetic resonance imaging; with i.v. contrast) or pre-sRT-CT (multislice computed tomography with i.v. and oral contrast) of the abdomen and pelvis assessed within 16 weeks prior to randomization.
5. WHO performance status 0–1 at randomization.
6. Age at randomization between 18 and 80 years.
7. Informed consent.

### Exclusion criteria

Patients with the following characteristics will be ineligible for this study.

1. Persistent PSA value 4–20 weeks after radical prostatectomy > 0.4 ng/ml.
2. Palpable mass in the prostatic fossa, unless histology proves no evidence of recurrence.
3. Pelvic lymph node enlargement >1 cm in short axis diameter of the abdomen and pelvis (cN1), unless the enlarged lymph node is sampled and negative.
4. Presence or history of bone metastases. Bone scan is mandatory in cases of clinical suspicion (e.g., bone pain).
5. Other malignancies within five years before planned sRT; non-melanoma skin cancers are allowed.
6. ADT or bilateral orchiectomy.
7. Previous pelvic radiotherapy.
8. Hip prosthesis.
9. Metal clusters/markers and patients with a pacemaker.
10. Severe or active co-morbidities impairing the feasibility of hyperthermia or dose intensified sRT including (but not exclusively limited to):
  chronic inflammatory bowel disease
  acute bacterial or fungal infection requiring intravenous antibiotics at the time of randomization
  unstable angina pectoris and/or congestive heart failure requiring hospitalization within the last 6 months
  transmural myocardial infarction within the last 6 months
  chronic obstructive pulmonary disease exacerbation or other respiratory disorders requiring hospitalization or precluding planned treatment within the study at the time of randomization
  psychiatric disorder precluding understanding of information on trial-related topics, giving informed consent or filling out QoL questionnaires
11. Concurrent treatment with other experimental drugs or other anti-cancer therapy; treatment in a clinical trial within 30 days prior to trial entry.

### Radiation treatment planning

Patients will be placed in the supine position with a comfortably filled bladder and empty rectum. The use of an endorectal balloon is allowed. Target volume definition will be performed in accordance with the European organisation for research and treatment of cancer (EORTC) guidelines [33]. Organs at risk (OAR) include bladder, rectum and femoral heads. The bladder wall (BW) and rectal wall (RW) are contoured using a 5 mm internal margin. Constraints for OAR are given in Table 1.

**Table 1:** Constraints for organs at risk (OARs) in the phase II study on radio-thermotherapy for biochemical recurrent prostate cancer

Organs at risk (OARs)	Constraints			
	V50	V60	V65	V70
Rectal wall	n.a.	≤50%	n.a.	≤20%
Bladder wall	n.a.	n.a.	≤50%	n.a.
Femoral heads	≤10%	n.a.	n.a.	n.a.

*n.a.* not applicable, *OAR* organs at risk, *V* organ volume receiving a specified dose

The definition of volumes and dose reporting will be in accordance with the International Commission on Radiation Units and Measurements (ICRU) Report 83 [34].

A modified treatment concept can be used in cases of clear identification of regions of risk (i.e., distinct localization of pT3a-region, R1-region, bed of seminal vesicles for pT3b-stages). In such cases, the standard target volume (EORTC-definition) will be treated to a total dose of 66.5 Gy (5 × 1.9 Gy/week) and the target volume for regions of risk (modified integrated boost volume) will be irradiated with 5 × 2.0 Gy/week to a total dose of 70.0 Gy.

### Salvage radiotherapy delivery

SRT is administered to a total dose of 70 Gy (35 fractions with single fractions of 2 Gy) over 7 weeks (modified treatment concept [see above] with reduced dose to the EORTC-target volume of 5x1.9 Gy/week to 66.5 Gy and an in integrated boost with 5x2.0 Gy/week to 70 Gy in regions with high risk of recurrence). SRT should be delivered once daily except at weekends. Intensity modulated RT (IMRT) or alternatively rotational techniques such as Rapidarc, Tomotherapy or volumetric modulated arc technique (VMAT) will be eligible.

### Concurrent hyperthermia

Regional hyperthermia will be carried out within 2 (max. within 4) hours after radiation treatment twice a week. Multichannel thermometry probes or MR-mapping temperature probes will be placed into the rectum and urethra/bladder and the thermal target temperature will be at least 41 °C for 60 min (maximum temperature: 43 °C). The total hyperthermia time is approximately 90 min including an induction period of 30 min. Ten hyperthermia sessions will be conducted. Hyperthermia should start in the first week of radiation treatment (see schedule in Fig. 1). Suboptimal hyperthermia sessions (time duration or temperature) are counted as hyperthermia session and will not be repeated. Due to any reason omitted hyperthermia sessions can be performed until the last fraction of radiation treatment. The interval between hyperthermia applications should be at least 48 h. Hyperthermia will be performed according to current guidelines [35, 36].

### Trial duration and follow-up assessment

The trial is planned to be initiated in summer 2015. The recruitment period of the entire study (100 patients) will take approximately 36 months. The primary endpoint of the study (acute toxicity) can be evaluated three months later. The follow-up period is three years. The whole study duration is estimated to be 75 months if the study is not terminated early (see criteria for termination). Follow-up visits are scheduled at 3 months after the end of treatment and 6-monthly thereafter, until the third year. Assessment includes overall survival, PSA-level, clinical recurrence-free survival with regard to local, regional and distant control. Time without treatment for prostate cancer and toxicity according to CTCAE 4.0 will be evaluated. QoL will be assessed at 3, 12, 24, 36 months after completion of treatment.

### Sample size calculation and criteria for termination of the study

The H0-hypothesis of the protocol is that ≥20 % of patients experience grade 3+ genitourinary and gastrointestinal acute toxicities (CTC v 4.0) or ≥30 % in case of pre-existing G2-toxicities.

Accordingly, the study is terminated if ≥20 % of patients (*n* = 10/50) experience grade 3+ genitourinary and gastrointestinal acute toxicities (CTC v 4.0) or ≥30 % in case of pre-existing G2-toxicities. If toxicity is below the mentioned threshold, the study will be continued to include 100 patients treated per protocol. In the final analysis (*n* = 100), the H0-hypothesis is rejected if ≤13 % (13/100) grade 3+ genitourinary and gastrointestinal acute toxicities (CTC v 4.0) were observed. This study (Simon’s design) has a significance level p < 0.05 and a power of 87.2 % if G3 + −toxicity is ≤10 %.

### Ethical and legal considerations

This study will be conducted in accordance with the guidelines of the local ethics committees. The protocol, patient information and informed consent sheets were submitted to the independent Ethics Committee of the Medical Faculty, University of Berlin, Germany and are submitted to the Ethics Committee of the Medical Faculty, University of Tuebingen, Germany. The “Independent Expert Committee of the DEGRO” stated that the dose range of 64–70 Gy as an application of therapeutic radiotherapy is within the frame of current health care. The study is in line with the Declaration of Helsinki, the laws and regulations of the country and in accordance with good clinical practice (ICH Harmonized Tripartite Guideline for Good Clinical Practice, http://www.ich.org/products/guidelines/efficacy/efficacy-single/article/good-clinicalpractice.html).

### Sponsorship

The study sponsor for patients treated at the Department of Radiation Oncology of the Charité Universitätsmedizin Berlin, Germany is the Charité Universitätsmedizin Berlin, Germany. The study sponsor for patients treated at the Department of Radiation Oncology of the University Hospital of Tuebingen is the University Hospital of Tuebingen, Germany.

## Results and discussion

This study will investigate the safety and feasibility of moderately dose-escalated radiotherapy combined with regional hyperthermia for patients with biochemical recurrence after prostatectomy. The applied radiation technique, including IMRT or rotational techniques, and thermometry (MR-based thermometry is allowed) of hyperthermia both represent the latest technical developments [37]. This ensures that the study is performed at the highest technical level and is essential to minimize potential modality-related side-effects, since knowledge on radio-thermotherapy after prostatectomy is limited [23].

All treatment optimization studies on sRT share the same local risks. Treatment is given to the urethral anastomosis where fibrosis, stenosis or, in the worst case, a leakage could occur. With regard to the most technically advanced randomized trial on postoperative radiotherapy using 3D-conformal radiotherapy (3D-CRT) to the prostatic fossa, the total dose of 60 Gy was associated with only 0.3 % G3+ side effects (1/307) [38]. Goldin et al. ascertained that there was no difference in toxicity between IMRT and 3D-CRT in the postoperative setting [39]. It might be speculated that this finding was based on the general low frequency of side effects in this radiation dose range and was independent of the radiation technique. At higher radiation dose levels, IMRT was beneficial for patients with definitive prostate radiotherapy. This was demonstrated in a propensity score-adjusted analyses (*n* = 12,976) of data from the Surveillance, Epidemiology, and End Results (SEER) database [40]. The use of IMRT compared with 3D-CRT was associated with less gastrointestinal morbidity and fewer hip fractures, but more erectile dysfunction potentially caused by the higher total dose in the IMRT group. The RTOG target volume definition [41] used in the Godin study was clearly larger compared to the EORTC target volume definition. The larger and more regular the treatment volume, the smaller the benefit from IMRT. Consequently, findings from trials using another target volume definition can only be applied with caution.

The SAKK 09/10-study did not detect significant differences in CTCAE-measured acute toxicity between 64 Gy and 70 Gy. Urinary quality of life was slightly but significantly worsened in the higher dose group, independent of radiation treatment technique [12]. Reduction of the high-dose target volume in cases of clear identification of high risk areas (modified boost concept) was reasonable and was therefore introduced in this study. A first hint on efficacy of radio-thermotherapy (secondary endpoint) could allow comparison of this study with the SAKK 09/10 outcome data, since target definition, cancer-related inclusion and exclusion criteria were equal. A recently published retrospective study demonstrated a significant benefit for patients with positive margin but no macroscopic recurrence treated to at least 70 Gy compared to a patient cohort with a total dose of 66 Gy (100 % vs. 66.7 %) [42].

The RTOG 96–01 study demonstrated that sRT to 64.8 Gy and 2 years of ADT increased PSA-control (57 % vs. 40 %) without any influence on overall or disease-specific survival at 7 years [11]. However, long-term ADT is associated with general side effects such as sarcopenia, erectile dysfunction, fatigue and osteoporosis with a risk of fractures [43]. Furthermore, there are hints that changes in lipid metabolism could lead to a greater incidence of diabetes and cardiovascular disease, including renal complications [44–46]. Consequently, Ahmadi et al. concluded that the best way of preventing side effects is to only use ADT when it is absolutely indicated [43]. Cardiovascular mortality did not differ in otherwise healthy study participants of 8 randomized trials on long-term ADT [47]. However, the influence of long-term-ADT in patients with pre-existing co-morbidities remains unclear. Therefore, hyperthermia could become a sRT optimization tool to circumvent long-term ADT and its suspected side-effects.

## Conclusion

The ongoing phase II study implements highly precise image-guided radiotherapy and regional hyperthermia. If the phase II study is found to be safe and feasible, a multicenter phase III study is planned to test whether the addition of ten hyperthermia applications to dose-intensified sRT improves biochemical control.
